# Supplementary material for: Unveiling the Bioactive Potential of the Invasive Jellyfish Phyllorhiza punctata Through Integrative Transcriptomic and Proteomic Analyses
Source: Biomolecules. 2025 Aug 4;15(8):1121. doi: 10.3390/biom15081121 (PMC12383608; doi:10.3390/biom15081121)
Supplement: Supplementary file 1 [file biomolecules-15-01121-s001.zip › Suplementary Material/FileS3/PEPFOLD4/XP_065055518_8898/clusters.html]

 PEP-FOLD clusters


Download archive of all models  here   

|  |  |  |
| --- | --- | --- |
| # | file | sOPEP |
| # cluster 1 - Size: 3 |  |  |
|  | XP\_065055518\_8898-model1 | -17.9274 |
|  | XP\_065055518\_8898-model1.1 | -14.1673 |
|  | XP\_065055518\_8898-model1.2 | -12.7548 |
| # cluster 2 - Size: 2 |  |  |
|  | XP\_065055518\_8898-model2 | -15.5173 |
|  | XP\_065055518\_8898-model2.1 | -13.1508 |
| # cluster 3 - Size: 16 |  |  |
|  | XP\_065055518\_8898-model3 | -15.2974 |
|  | XP\_065055518\_8898-model3.1 | -13.9923 |
|  | XP\_065055518\_8898-model3.2 | -13.7553 |
|  | XP\_065055518\_8898-model3.3 | -13.5757 |
|  | XP\_065055518\_8898-model3.4 | -13.4875 |
|  | XP\_065055518\_8898-model3.5 | -13.3775 |
|  | XP\_065055518\_8898-model3.6 | -13.32 |
|  | XP\_065055518\_8898-model3.7 | -13.198 |
|  | XP\_065055518\_8898-model3.8 | -13.1336 |
|  | XP\_065055518\_8898-model3.9 | -13.0097 |
|  | XP\_065055518\_8898-model3.10 | -13.0071 |
|  | XP\_065055518\_8898-model3.11 | -13.0021 |
|  | XP\_065055518\_8898-model3.12 | -12.9639 |
|  | XP\_065055518\_8898-model3.13 | -12.7982 |
|  | XP\_065055518\_8898-model3.14 | -12.7752 |
|  | XP\_065055518\_8898-model3.15 | -12.7667 |
| # cluster 4 - Size: 7 |  |  |
|  | XP\_065055518\_8898-model4 | -14.8096 |
|  | XP\_065055518\_8898-model4.1 | -14.0904 |
|  | XP\_065055518\_8898-model4.2 | -13.619 |
|  | XP\_065055518\_8898-model4.3 | -13.4086 |
|  | XP\_065055518\_8898-model4.4 | -13.1868 |
|  | XP\_065055518\_8898-model4.5 | -12.8156 |
|  | XP\_065055518\_8898-model4.6 | -12.7235 |
| # cluster 5 - Size: 1 |  |  |
|  | XP\_065055518\_8898-model5 | -14.8047 |
| # cluster 6 - Size: 2 |  |  |
|  | XP\_065055518\_8898-model6 | -14.6974 |
|  | XP\_065055518\_8898-model6.1 | -13.5169 |
| # cluster 7 - Size: 10 |  |  |
|  | XP\_065055518\_8898-model7 | -14.6747 |
|  | XP\_065055518\_8898-model7.1 | -13.877 |
|  | XP\_065055518\_8898-model7.2 | -13.7113 |
|  | XP\_065055518\_8898-model7.3 | -13.658 |
|  | XP\_065055518\_8898-model7.4 | -13.6333 |
|  | XP\_065055518\_8898-model7.5 | -13.5529 |
|  | XP\_065055518\_8898-model7.6 | -13.4618 |
|  | XP\_065055518\_8898-model7.7 | -13.3788 |
|  | XP\_065055518\_8898-model7.8 | -12.9808 |
|  | XP\_065055518\_8898-model7.9 | -12.873 |
| # cluster 8 - Size: 21 |  |  |
|  | XP\_065055518\_8898-model8 | -14.3546 |
|  | XP\_065055518\_8898-model8.1 | -14.1258 |
|  | XP\_065055518\_8898-model8.2 | -13.7549 |
|  | XP\_065055518\_8898-model8.3 | -13.7359 |
|  | XP\_065055518\_8898-model8.4 | -13.6822 |
|  | XP\_065055518\_8898-model8.5 | -13.6091 |
|  | XP\_065055518\_8898-model8.6 | -13.6088 |
|  | XP\_065055518\_8898-model8.7 | -13.5967 |
|  | XP\_065055518\_8898-model8.8 | -13.5787 |
|  | XP\_065055518\_8898-model8.9 | -13.3684 |
|  | XP\_065055518\_8898-model8.10 | -13.3678 |
|  | XP\_065055518\_8898-model8.11 | -13.3385 |
|  | XP\_065055518\_8898-model8.12 | -13.3093 |
|  | XP\_065055518\_8898-model8.13 | -13.2377 |
|  | XP\_065055518\_8898-model8.14 | -13.204 |
|  | XP\_065055518\_8898-model8.15 | -13.1245 |
|  | XP\_065055518\_8898-model8.16 | -13.1033 |
|  | XP\_065055518\_8898-model8.17 | -12.9964 |
|  | XP\_065055518\_8898-model8.18 | -12.9739 |
|  | XP\_065055518\_8898-model8.19 | -12.8562 |
|  | XP\_065055518\_8898-model8.20 | -12.732 |
| # cluster 9 - Size: 4 |  |  |
|  | XP\_065055518\_8898-model9 | -14.2726 |
|  | XP\_065055518\_8898-model9.1 | -13.5361 |
|  | XP\_065055518\_8898-model9.2 | -12.9687 |
|  | XP\_065055518\_8898-model9.3 | -12.8239 |
| # cluster 10 - Size: 1 |  |  |
|  | XP\_065055518\_8898-model10 | -13.8783 |
| # cluster 11 - Size: 5 |  |  |
|  | XP\_065055518\_8898-model11 | -13.4442 |
|  | XP\_065055518\_8898-model11.1 | -13.3752 |
|  | XP\_065055518\_8898-model11.2 | -13.0969 |
|  | XP\_065055518\_8898-model11.3 | -13.0492 |
|  | XP\_065055518\_8898-model11.4 | -12.6598 |
| # cluster 12 - Size: 4 |  |  |
|  | XP\_065055518\_8898-model12 | -13.3373 |
|  | XP\_065055518\_8898-model12.1 | -13.0996 |
|  | XP\_065055518\_8898-model12.2 | -12.9468 |
|  | XP\_065055518\_8898-model12.3 | -12.803 |
| # cluster 13 - Size: 1 |  |  |
|  | XP\_065055518\_8898-model13 | -13.3249 |
| # cluster 14 - Size: 2 |  |  |
|  | XP\_065055518\_8898-model14 | -13.3218 |
|  | XP\_065055518\_8898-model14.1 | -13.1261 |
| # cluster 15 - Size: 7 |  |  |
|  | XP\_065055518\_8898-model15 | -13.3134 |
|  | XP\_065055518\_8898-model15.1 | -13.0115 |
|  | XP\_065055518\_8898-model15.2 | -13.0088 |
|  | XP\_065055518\_8898-model15.3 | -13.0075 |
|  | XP\_065055518\_8898-model15.4 | -12.8136 |
|  | XP\_065055518\_8898-model15.5 | -12.8129 |
|  | XP\_065055518\_8898-model15.6 | -12.8044 |
| # cluster 16 - Size: 3 |  |  |
|  | XP\_065055518\_8898-model16 | -13.2518 |
|  | XP\_065055518\_8898-model16.1 | -13.0091 |
|  | XP\_065055518\_8898-model16.2 | -12.9186 |
| # cluster 17 - Size: 3 |  |  |
|  | XP\_065055518\_8898-model17 | -13.1795 |
|  | XP\_065055518\_8898-model17.1 | -13.1294 |
|  | XP\_065055518\_8898-model17.2 | -12.8839 |
| # cluster 18 - Size: 1 |  |  |
|  | XP\_065055518\_8898-model18 | -13.0748 |
| # cluster 19 - Size: 2 |  |  |
|  | XP\_065055518\_8898-model19 | -13.0475 |
|  | XP\_065055518\_8898-model19.1 | -12.7033 |
| # cluster 20 - Size: 1 |  |  |
|  | XP\_065055518\_8898-model20 | -13.0435 |
| # cluster 21 - Size: 1 |  |  |
|  | XP\_065055518\_8898-model21 | -12.9756 |
| # cluster 22 - Size: 3 |  |  |
|  | XP\_065055518\_8898-model22 | -12.8969 |
|  | XP\_065055518\_8898-model22.1 | -12.7548 |
|  | XP\_065055518\_8898-model22.2 | -12.6775 |
|  |  |  |
